# Supplementary material for: miR-9 and miR-181a Target Gab2 to Inhibit the Proliferation and Migration of Hepatocellular Carcinoma HepG2 Cells
Source: Genes (Basel). 2022 Nov 18;13(11):2152. doi: 10.3390/genes13112152 (PMC9690539; doi:10.3390/genes13112152)
Supplement: Supplementary file 1 [file genes-13-02152-s001.zip › Supplemental Figure S1.pdf]

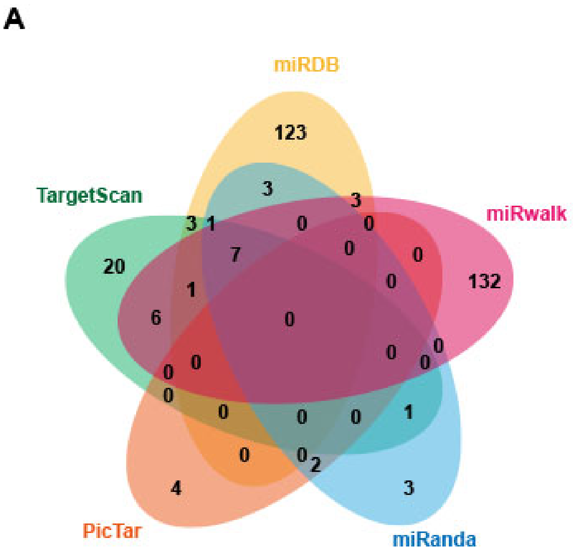

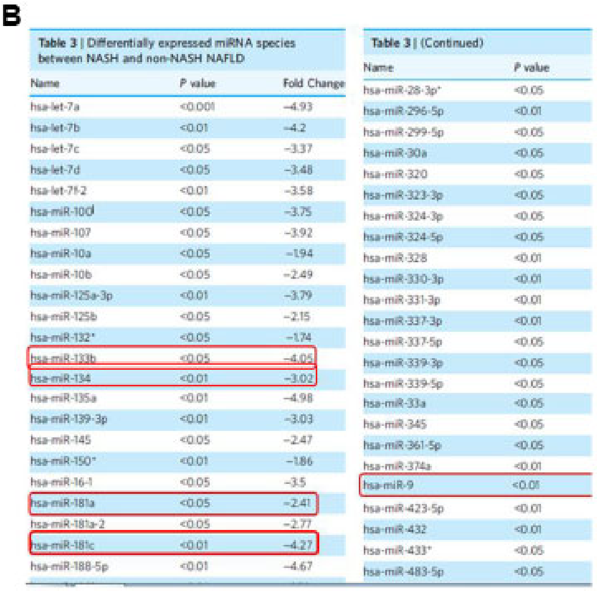

B

Table 3 | Differentially expressed miRNA species between NASH and non-NASH NAFLD

Name	P value	Fold Change
hsa-let-7a	<0.001	−4.93
hsa-let-7b	<0.01	−4.2
hsa-let-7c	<0.05	−3.37
hsa-let-7d	<0.05	−3.48
hsa-let-7f-2	<0.01	−3.58
hsa-miR-100[d]	<0.05	−3.75
hsa-miR-107	<0.05	−3.92
hsa-miR-10a	<0.05	−1.94
hsa-miR-10b	<0.05	−2.49
hsa-miR-125a-3p	<0.01	−3.79
hsa-miR-125b	<0.05	−2.15
hsa-miR-132*	<0.05	−1.74
hsa-miR-133b	<0.05	−4.05
hsa-miR-134	<0.01	−3.02
hsa-miR-135a	<0.01	−4.98
hsa-miR-139-3p	<0.01	−3.03
hsa-miR-145	<0.05	−2.47
hsa-miR-150*	<0.01	−1.86
hsa-miR-16-1	<0.05	−3.5
hsa-miR-181a	<0.05	−2.41
hsa-miR-181a-2	<0.05	−2.77
hsa-miR-181c	<0.01	−4.27
hsa-miR-188-5p	<0.01	−4.67

Table 3 | (Continued)

Name	P value
hsa-miR-28-3p*	<0.05
hsa-miR-296-5p	<0.01
hsa-miR-299-5p	<0.05
hsa-miR-30a	<0.05
hsa-miR-320	<0.05
hsa-miR-323-3p	<0.05
hsa-miR-324-3p	<0.05
hsa-miR-324-5p	<0.05
hsa-miR-328	<0.01
hsa-miR-330-3p	<0.01
hsa-miR-331-3p	<0.01
hsa-miR-337-3p	<0.01
hsa-miR-337-5p	<0.05
hsa-miR-339-3p	<0.05
hsa-miR-339-5p	<0.05
hsa-miR-33a	<0.05
hsa-miR-345	<0.05
hsa-miR-361-5p	<0.05
hsa-miR-374a	<0.01
hsa-miR-9	<0.01
hsa-miR-423-5p	<0.01
hsa-miR-432	<0.01
hsa-miR-433*	<0.05
hsa-miR-483-5p	<0.05

Supplemental Figure S1. Prediction of GAB2-targeting miRNAs. (A) Prediction of GAB2-targeting miRNAs by five bioinformatics websites. **(B)** the abnormally expressed miRNAs in fatty liver and hepatocellular carcinoma.
