# Supplementary material for: miR-9 and miR-181a Target Gab2 to Inhibit the Proliferation and Migration of Hepatocellular Carcinoma HepG2 Cells
Source: Genes (Basel). 2022 Nov 18;13(11):2152. doi: 10.3390/genes13112152 (PMC9690539; doi:10.3390/genes13112152)
Supplement: Supplementary file 1 [file genes-13-02152-s001.zip › Supplemental Figure S2.pdf]

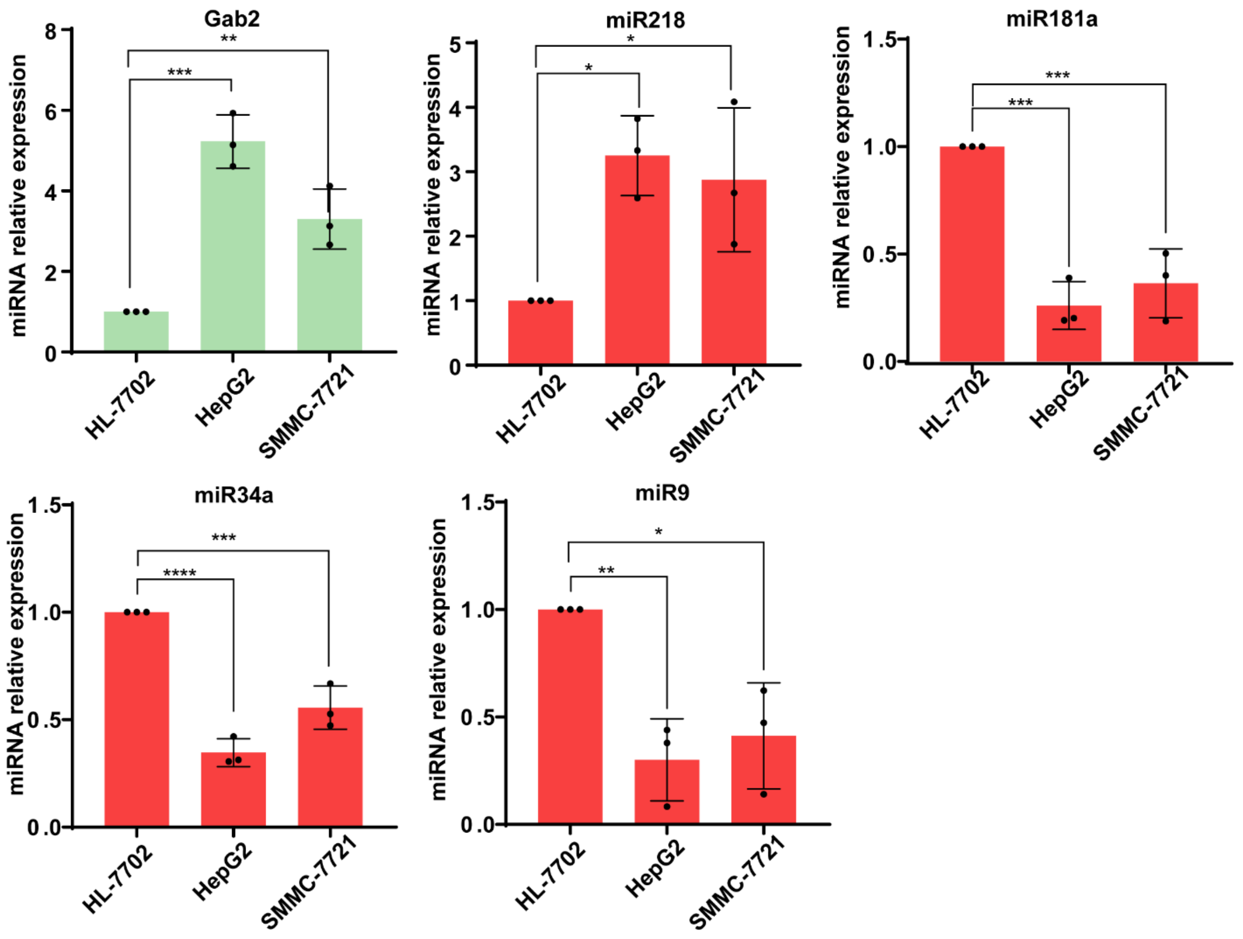

Supplemental Figure S2. Expression of miRNAs in normal liver cell HL-7702 and hepatocellular carcinoma cell line HepG2 and SMMC-7721. The results are presented as mean ± SD. Statistical comparisons of values were made using Student's t-test. ns, no significance; * $p < 0.05$, ** $p < 0.01$, *** $p < 0.001$.
