# Supplementary material for: miR-9 and miR-181a Target Gab2 to Inhibit the Proliferation and Migration of Hepatocellular Carcinoma HepG2 Cells
Source: Genes (Basel). 2022 Nov 18;13(11):2152. doi: 10.3390/genes13112152 (PMC9690539; doi:10.3390/genes13112152)
Supplement: Supplementary file 1 [file genes-13-02152-s001.zip › Supplemental File S1.pdf]

The primer for Gab2 mRNA:
Forward primer: CCCTGTGTCAAACCACATGC
Reverse primer: CTGCTCTTCGGCTTATGCACT

The mimic and inhibitors of miRNA:
miR-9-5p mimic 5-UCUUUGGUUAUCUAGCUGUAUGA-3
3-AGAAACCAAUAGAUCGACAUACU-5
inhibitor 5-UCAUACAGCUAGAUAACCAAAGA-3

miR-34a-5p mimic: 5-UGGCAGUGUCUUAGCUGGUUGU-3
3-ACCGUCACAGAAUCGACCAACA-5
Inhibitor: 5-ACAACCAGCUAAGACACUGCCA-3

miR-181a-5p mimic: 5-AACAUUCAACGCUGUCGGUGAGU-3
3-UUGUAAGUUGCGACAGCCACUCA-5
Inhibitor: 5-ACUCACCGACAGCGUUGAAUGUU-3

miR-181c-5p mimic: 5-AACAUUCAACCUGUCGGUGAGU-3
3-UUGUAAGUUGCACAGCCACUCA-5
Inhibitor: 5-ACUCACCGACACGUUGAAUGUU-3

The primers of miRNA-Gab2 3'UTR binding reporter-luciferase constructs:
miR-181a-Forward primer:AGCTTTGTTTAAACGGACCGTTCCTAGGATGG
miR-181a-Reverse primer:GCTCTAGAAATGCTAGGAGGAGAGGTGCAGCT
miR-9-Forward primer:CTAGCTAGCTCGGGGCCCAGCTGTGATTTTT
miR-9-Reverse primer:ACGCGTCGACACAGTGTCCTTGTCATCTCCA

The mutation primers were designed as follows:
miR-181-a-Fm: GGGTGGGGGATCCACCCACCTTTATGTTGACTGTT
miR-181-a-Rm: AACAGTCAACATAAAGGTGGGTGGATCCCCCACCC
miR-9-Fm: AGGGAGGAGCGGATCCTCACGTTGCCTTGGATAAT
miR-9-Rm: ATTATCCAAGGCAACGTGAGGATCCGCTCCTCCCT
